# Special Issue “Using Model Organisms to Study Complex Human Diseases”

**DOI:** 10.3390/ijms27125449

**Published:** 2026-06-16

**Authors:** Roberto Piergentili

**Affiliations:** Istituto di Biologia e Patologia Molecolari (IBPM) del Consiglio Nazionale delle Ricerche (CNR), at Dipartimento di Biologia e Biotecnologie, Università Sapienza di Roma, Piazzale Aldo Moro 5, 00185 Rome, Italy; roberto.piergentili@cnr.it

A model organism is defined as a living species that allows the study of specific biological problems, where discoveries may be applied—with a limited number of adjustments—to a wide range of other species. To be classified as a “model,” an organism should possess specific attributes: it must be relatively simple, be possible to establish in numerous laboratories, offer rapid generation times, and have a relatively low rearing cost, such as Drosophila melanogaster [1]. Furthermore, these organisms should exhibit stable and easy-to-recognize phenotypes, allow for study at the “-omics” level, and have specific tools available for studying the modification of gene expression and performing loss- and gain-of-function experiments [2,3]. In fact, one of the primary strengths of model organisms lies in their experimental tractability. Many model systems are characterized by features such as short generation times and high reproductive rates, which collectively enable rapid and cost-effective experimentation. These attributes facilitate large-scale genetic and pharmacological screenings, as well as precise manipulation of gene function through contemporary genome-editing approaches. Moreover, the availability of standardized protocols and well-annotated genomic resources enhances reproducibility and comparability across laboratories, thereby strengthening the robustness of experimental findings.

The spectrum of model organisms is vast, ranging from unicellular eukaryotes to sophisticated multicellular models. Classical model organisms include prokaryotes such as the bacterium *Escherichia coli* and eukaryotes, both unicellular (the budding yeast *Saccharomyces cerevisiae*, the fission yeast *Schizosaccharomyces pombe*, or numerous human/mammalian cell cultures used in cancer research) and multicellular. Multicellular examples include the roundworm *Caenorhabditis elegans*, the fruit fly *Drosophila melanogaster*, the zebrafish *Danio rerio*, the plant *Arabidopsis thaliana*, the African clawed frog *Xenopus laevis*, the rat *Rattus norvegicus*, and the mouse *Mus musculus*. Unicellular models are particularly adept at elucidating fundamental cell division and metabolic pathways, while multicellular models can recapitulate systemic human pathologies. Additional models have been developed over time that are evolutionarily distant from one another to overcome the limitations of working on overly simplified models.

Model organisms have long constituted a foundational pillar of biomedical research, serving as indispensable tools in investigating the molecular and cellular mechanisms underlying human disease. Their utility is grounded in the principle of evolutionary conservation, whereby fundamental biological pathways are shared across species, enabling experimental findings obtained in non-human systems to inform our understanding of human physiology and pathology [4,5]. In the contemporary era of precision medicine, deciphering the intricate molecular etiologies underlying complex human diseases remains a paramount challenge, and while high-throughput multi-omics approaches and artificial intelligence continue to revolutionize clinical diagnostics, the mechanistic validation of these data still relies heavily on the use of model organisms, which bridge the gap between in silico predictions and in vivo physiological relevance. The integration of advanced genomic, transcriptomic, and gene-editing technologies has reinforced the relevance of these organisms in translational research, especially regarding disease mechanisms and therapeutic target identification. The remarkable evolutionary conservation of core biochemical networks ensures that genetic and pharmacological insights gleaned from these non-human species can be robustly translated into therapeutic strategies for inherited disorders, rare diseases, and oncology [6]. From this perspective, model organisms provide controlled environments for hypothesis testing, allowing for invasive and longitudinal investigations that would be ethically and practically infeasible in human studies. This experimental flexibility permits the dissection of causal relationships between genetic, environmental, and physiological variables. Furthermore, they serve as essential intermediates in the translational pipeline, bridging the gap between in vitro studies and clinical applications by allowing for the preclinical evaluation of therapeutic strategies. The conservation of basic cellular functions—such as DNA replication and repair, transcription, translation, post-transcriptional regulation, epigenetics, and physiology—makes these organisms amenable for studying the etiology of human pathology.

Despite their strengths, the use of model organisms is associated with significant limitations [7,8]. Foremost is the issue of interspecies differences; while many biological pathways are conserved, divergences in physiology, immune responses, and metabolic processes may limit the extent to which findings can be extrapolated to humans. These discrepancies contribute to challenges in translational success, where promising preclinical results may fail to replicate in clinical settings. Additionally, the inherent reductionism of model systems may oversimplify complex, multifactorial human diseases. Standardization and biological variability also influence outcomes. While inbred strains minimize variability, they may fail to capture the genetic diversity observed in human populations, limiting the generalizability of findings. Ethical considerations represent another critical factor, as the use of animal models raises concerns regarding animal welfare and necessitates strict regulatory oversight. Economic and logistical constraints, including the costs of maintaining animal facilities, can also limit accessibility. These challenges have prompted increasing interest in alternative or complementary approaches, such as in vitro systems and computational modeling. Nevertheless, at present, model organisms remain irreplaceable foundations in the continuum of translational medicine, despite their limitations necessitate a cautious and integrative approach. Future progress will likely depend on combining model organisms with emerging human-relevant systems to foster a more comprehensive understanding of disease biology.

This Special Issue, titled ‘Using Model Organisms to Study Complex Human Diseases’, brings together cutting-edge research that leverages these diverse platforms to expand our understanding of human physiopathology. This Special Issue features ten publications, which deal with diverse human conditions. The porcine model (*Sus scrofa*) has been used in three manuscripts, while other models studied in these contributions include cultured stem cells (two contributions), *R. norvegicus*, *C. elegans*, *M. musculus*, and *Microtus ochrogaster* (monogamous prairie voles). Finally, a review is included regarding the use of invertebrate and vertebrate animal models for the study of Down Syndrome, where the author illustrates how models developed in *C. elegans*, *D. melanogaster*, *D. rerio* and *M. musculus* helped (and still help) scientists to better understand this extremely complex condition.

Interested readers may found all the contributions at the following link: https://www.mdpi.com/journal/ijms/special_issues/PICFUH255A. We are grateful to all authors who submitted their work and supported this collection and to the *International Journal of Molecular Sciences* staff, whose help was invaluable in the success of this editorial project. All articles are open access to readers and distributed under the terms and conditions of the Creative Commons Attribution (CC BY) license 4.0.
